# Cyberbullying and internet gaming disorder in Chinese youth: The role of positive youth development attributes

**DOI:** 10.3389/fpubh.2022.1017123

**Published:** 2022-11-21

**Authors:** Guo-Xing Xiang, Yan-Hong Zhang, Xiong Gan, Ke-Nan Qin, Ya-Ning Zhou, Min Li, Xin Jin

**Affiliations:** ^1^Department of Psychology, College of Education and Sports Sciences, Yangtze University, Jingzhou, China; ^2^Department of Psychology, Yangtze University College of Technology and Engineering, Jingzhou, China

**Keywords:** positive youth development attributes, cyberbullying, internet gaming disorder, mediating effect, Chinese adolescents

## Abstract

**Introduction:**

As digital natives, young people enjoy the convenience and benefits of the internet but also suffer from unique developmental problems of this age, such as cyberbullying and internet gaming disorder (IGD). Research suggests that these online problem behaviors enjoy high prevalence and various negative impacts. To prevent or intervene, this study attempts to explore the association between cyberbullying and IGD and the potential protectors from the positive youth development (PYD) perspective.

**Methods:**

Through the convenience sampling method, a sample of 463 Chinese adolescents was recruited and participated in the survey. They completed a questionnaire regarding PYD attributes, cyberbullying, IGD, and demographic information.

**Results:**

After controlling adolescents' sex and age, results of regression analyses indicated that cyberbullying was positively associated with IGD; PYD attributes had negative cumulative effects on cyberbullying and IGD; and cyberbullying and IGD were negatively related to PYD attributes. Moreover, the mediating effect of PYD attributes was significant in the relationship between cyberbullying and IGD.

**Discussion:**

Specifically, it is very possible for adolescents who have experienced one online problem behavior to suffer from another one. Fortunately, positive personal attributes could effectively buffer this cascading effect. These findings may provide theoretical and practical guidance for practitioners that improving PYD attributes may be a promising approach to prevent or reduce adolescent cyberbullying and IGD.

## Introduction

In the digital era, while enjoying the convenience and resources brought by the development of the internet and mobile devices, individuals are also faced with many unique risks of this age. Considerable new types of developmental problems have emerged among young generations in recent decades, such as cyberbullying and internet gaming disorder (IGD). Cyberbullying is conceptualized as “using information and communication technologies to repeatedly and intentionally harm, harass, hurt, and/or embarrass a target” (1). The prevalence rate of cyberbullying (preparation and victimization) ranged from 6.0 to 57.5% among worldwide children and adolescents (2). Besides, IGD refers to uncontrollable, excessive, and compulsive use of internet games, leading to social and emotional problems (3). The pooled prevalence of IGD among worldwide adolescents was 4.6%, while its prevalence in Chinese adolescents was 13.0% (4, 5). Previous studies have indicated that adolescents who engage in cyberbullying and IGD tend to suffer from more psychological disorders and behavioral problems (e.g., depression, posttraumatic stress symptoms, and non-suicidal self-injury) (6–8). Based on the above prevalence and adverse effects, this study aims to investigate the potential protectors that could prevent adolescents from cyberbullying and IGD.

### Cyberbullying and IGD

Under this background, scholars have paid much attention to further explaining cyberbullying and IGD among adolescents, and their contributions indeed enrich the understanding of these phenomena. Scholars have revealed that family factors (e.g., function and conflict), school factors (e.g., climate and student–teacher connectedness), and personal factors (e.g., self-control and personal growth initiative) can explain these online problem behaviors in youth (9–12). However, little research has noticed the interaction between cyberbullying and IGD among adolescents. According to the developmental cascade theory, the development of various systems will affect each other, and the development of different factors in the same system will affect each other as well (13). Cyberbullying and IGD can be recognized as two common types of online behavioral problems. If an individual experiences cyberbullying, it may be more likely for him or her to suffer from IGD, and vice versa. Considerable indirect evidence has revealed the potential interaction between cyberbullying and IGD. For instance, two longitudinal studies suggested that internet addiction positively predicted cyberbullying half a year later among adolescents, and vice versa (14, 15). In China, Liu et al. (16) reported that adolescents who were bullied online tended to be addicted to the internet half a year later. Moreover, numerous studies indicated that Chinese adolescents who were bullied offline were more likely to suffer from IGD (17, 18). Rao et al. (19) suggested that IGD was associated with increased odds of both cyberbullying perpetration and victimization among Chinese adolescents. In summary, it is very common and easy for adolescents who have experienced IGD to engage in cyberbullying, and vice versa. Correspondingly, those adolescents may suffer from more cumulative adverse effects triggered by these online behavioral problems. Therefore, it is necessary to focus on the cascading effect between cyberbullying and IGD and explore the potential buffering factors.

### PYD attributes as a mediator

When thinking about protective and positive factors in youth, scholars can never ignore the positive youth development theory (PYD). It underlines that all young people do have strengths and potential to promote their flourishing and prevent negative development outcomes (20). From this perspective, various PYD modes are developed to summarize the positive attributes, including 5Cs, 6Cs, 7Cs, Catalano's 15-attribute PYD models, and developmental assets (21, 22). Despite these various forms, these models share a core assumption that the more positive attributes one has, the more likely he or she will develop more positive outcomes and fewer problems (e.g., cyberbullying and IGD) (20). In China, most scholars applied Catalano's 15-attribute PYD model to investigate adolescents' development (23, 24). In this model, 15 attributes include bonding, resilience, social competence, emotional competence, cognitive competence, behavioral competence, moral competence, self-determination, self-efficacy, spirituality, beliefs in the future, clear and positive identity, prosocial involvement, prosocial norms, and recognition for positive behavior (22). Prior literature has repeatedly confirmed the effects of PYD attributes on individual growth in various cultures (21, 25–27). Moreover, these positive factors are also confirmed to protect young people from developmental problems during the COVID-19 pandemic (24, 28). However, the function of these PYD models has rarely been evaluated in digital contexts, whereas today's young generations are digital natives who have grown up in the digital age and naturally use the digital media (29). Furthermore, Navarro and Tudge (30) also notice the influence of technological and virtual contexts on human development and include them in the neo-ecological theory, which highlights that, as with the physical environment, digital settings will also shape individual growth in the current and following centuries. Accordingly, this study attempts to examine the protective role of PYD attributes in the cascading effect between cyberbullying and IGD.

Based on the shared core assumption of PYD theory, adolescents who have obtained more positive attributes are less likely to suffer from cyberbullying and IGD (20). Existing literature has provided both indirect and direct evidence to support this inference. As for cyberbullying or traditional bullying, Schiamberg et al. (31) proposed a new approach incorporating PYD theory to investigate and intervene in offline and online bullying behaviors. Specifically, bonding, resilience, social competence, emotional competence, moral competence, and clear and positive identity (e.g., self-esteem) were confirmed to significantly predict cyberbullying behaviors among young students (32–35). Moreover, personal resources and several PYD attributes were significantly associated with bullying behaviors among adolescents (36, 37). In terms of IGD, previous studies have revealed the protective effects of various developmental assets in reducing Chinese adolescents' IGD (28, 38). Besides, resilience, social competence, emotional competence (e.g., emotional intelligence), self-efficacy, and self-determination are negatively associated with IGD in young people (39–42). As a complement, scholars have claimed that PYD attributes are effective protectors against social networking addiction and internet addiction in Chinese youth (43, 44). Overall, adolescents who develop more PYD attributes tend to experience less cyberbullying and IGD.

In addition, according to the risk-buffering model, PYD attributes may serve as a buffering factor that could alleviate the risk linkage between cyberbullying and IGD (45). Existing research has examined the mediating effect of PYD attributes on the associations between internet addiction and depression, family functioning and internalizing problems, and family functioning and delinquency in Chinese adolescents (46–48). Moreover, scholars also revealed the mediating effects of the 5Cs and internal developmental assets on the relation between school empowerment and school satisfaction and the association between family dinner and happiness in adolescents, respectively (49, 50). Considering the above literature review on the associations between PYD attributes, cyberbullying, and IGD, as well as the mediating effect of PYD attributes, it is reasonable to assume that PYD attributes could also function as a mediator between cyberbullying and IGD among Chinese adolescents.

### Current study

To sum up, despite the rich achievements in the study of cyberbullying and IGD among adolescents, there are still several research gaps in the existing literature, which the current study attempts to address. First, the interaction between cyberbullying and IGD has been neglected, and more attention has been paid to separately investigating them. However, according to the developmental cascade theory, cyberbullying and IGD are two specific types of online behavioral problems whereby they may affect each other and lead to cumulative adverse effects (13). Therefore, the first purpose of this study is to examine the association between cyberbullying and IGD. Second, previous research has paid limited attention to exploring the protective factors of these two online problem behaviors, while risk factors are favored by existing literature. Moreover, the limited literature including positive factors only investigates single or a few factors that may protect adolescents from these problems. Hence, the current study, based on the PYD theory (20), adopts the Catalano's 15 PYD attributes model to evaluate its cumulative and protective effects on cyberbullying and IGD. Third, it is not difficult to realize that most studies concerning PYD attributes consider PYD attributes as antecedents of development outcomes, which may provide guidance for related intervention from a precautionary perspective. But from the perspective of making amends, this study takes PYD attributes as a buffer to assess whether PYD attributes could prevent the effects of one existing problem behavior on the other one. To contribute to these research gaps, the current study considers the theoretical and empirical reviews on the associations between cyberbullying, IGD, and PYD attributes among adolescents and hypothesizes that:

***Hypothesis 1:*** *Cyberbullying will be positively associated with IGD*.

***Hypothesis 2:*** *PYD attributes may have cumulative effects on cyberbullying and IGD*.

***Hypothesis 3:*** *Cyberbullying and IGD may be negatively associated with PYD attributes*.

***Hypothesis 4:*** *PYD attributes will mediate the relationship between cyberbullying and IGD*.

## Method

### Participants and procedure

Participants were recruited from seven public middle and high schools in Shaanxi, Sichuan, and Hubei provinces, Mainland China, through the convenience sampling method. These three provinces are representative regions of the Northwest, Southwest, and Central China, respectively. Participants without dyslexia, with normal (corrected) vision, and able to write normally were allowed to participate in the survey. Totally, 463 adolescents participated in the data collection and completed a paper-and-pen questionnaire. Among them, 89 were from Shaanxi province, 171 were from Sichuan province, and 203 were from Hubei province. After excluding some students who incompletely responded and/or same responded to more than half of the items, the final sample includes 425 adolescents (*M*_*age*_ = 15.06 years, *SD*_*age*_ = 1.48, *Rang*_*age*_: 11–18, 246 males). Among them, there are 115 middle school students and 310 high school students. 26.59% of the adolescents are from only-child families, and 79.29% are from families at the average economic level in their provinces.

From November, 2020, to January, 2021, adolescents completed a self-reported questionnaire. Prior to the formal data collection, written consent was obtained from the school leaders, parents, and adolescents by explaining the detailed content of the survey. Well-trained researchers conducted the survey within 40 min during school hours. Honest responses were encouraged by informing them of the anonymity of the survey and their right to quit at any time. Moreover, adolescents were informed of the voluntary nature of the survey and they did not receive any kind of gift for their participation. The Research Ethics Committee of the College of Education and Sports Sciences at Yangtze University provided ethical approval for the whole procedures in the current study.

### Measures

#### Cyberbullying/victimization

The Chinese version of the E-Bullying and E-Victimization Scale (E-BVS) was employed to measure individuals' involvement in online bullying and victimization in the past week (51). The E-BVS has two subscales, and each subscale has six items. Responses to these 12 items are rated on a 7-point Likert scale, ranging from 0 (never) to 6 (6 times or more). One example item is “How many times did you make up something about someone to make others not like him/her anymore using emails, texting, short messages, on a website such as Renren, etc.”. A higher total score indicates a higher involvement level of cyberbullying and victimization. This scale shows good reliability and validity among Chinese adolescents (51). In the current sample, the Cronbach's αs of the total scale, cyberbullying subscale, and cybervictimization subscale were 0.920, 0.908, and 0.885, respectively.

#### Internet gaming disorder

The Chinese version of the Internet Gaming Disorder Questionnaire (IGDQ) was adopted to assess adolescents' IGD over the past half year (52). The IGDQ includes 11 items, and one example is “Have you ever done poorly on a school assignment or test because you spent too much time playing internet games?”. Participants are asked to respond on a 3-point Likert scale ranging from 1 (never) to 3 (often). A higher total score indicates a higher level of IGD. This scale suggests good reliability and validity in Chinese adolescent samples (52). In the current sample, its Cronbach's α was 0.858.

#### Positive youth development attributes

The Chinese version of the Positive Youth Development Scale (CPYDS) was adopted to assess the quality and quantity of adolescents' PYD attributes (22). The CPYDS composes 90 items, and one example item is “My belief is that even though tomorrow will become worse, I will still live in a good manner”. This scale measures 15 kinds of positive attributes such as social competence, behavioral competence, and spirituality. Response options are rated on a 6-point Likert scale ranging from 1 (not at all or rarely) to 6 (extremely or almost always). A higher total score reflects more types and higher levels of PYD attributes. The CPYDS demonstrates good reliability and validity in Chinese youth samples (24). In the current study, the Cronbach's α of this scale was 0.963.

#### Demographic covariates

Several demographic factors were assessed in this study, including adolescent age, gender (1 = male, 2 = female), grade, only-child family (1 = yes, 2 = no), and family economic level (1 = above the average level, 2 = at the average level, 3 = below the average level).

### Analytic plan

First, the data was collected through the questionnaire method, so it is necessary to statistically assess the potential common method bias. We employed the Harman's single factor test to examine it in all research items prior to the formal analyses. Second, descriptive statistics were adopted to calculate the means, standard deviations, skewness, and kurtosis of the main variables. Third, Pearson correlation analysis was conducted to evaluate the bivariate associations for the main variables in this study. Fourth, model 4 in PROCESS macro was used to test the mediating effects of PYD attributes between cyberbullying and IGD. Specifically, after examining the mediating effects between total involvement in cyberbullying and IGD, the mediation effects were further assessed in the associations between cyberbullying perpetration, cyberbullying victimization, and IGD. The bootstrapping method was used to generate 95% bias-corrected confidence intervals (CI) for the indirect effects in these models based on 5,000 resamples, and the 95% CIs excluding zero represent statistically significant. In these mediation models, adolescents' sex and age were considered as control variables. Moreover, scores of each variable were converted to standardized Z-scores to avoid multicollinearity problems. All analytic procedures were performed *via* SPSS 26.0 and the PROCESS macro.

## Results

### Common method bias

The results of Harman's single factor test indicated 26 distinct factors with eigenvalues greater than one, and the largest factor accounted for 24.51% of the total variance, which is less than the threshold level of 40%. Therefore, the common method variance was restricted in the current data set.

### Descriptive and correlational analyses

Table 1 presents the means, standard deviations, skewness, kurtosis, and bivariate correlations of the main variables. As expected, PYD attributes were negatively associated with IGD and three indicators of cyberbullying (*rs* < 0, *ps* < 0.01). Adolescents who obtained more PYD attributes were less prone to being involved in online problem behaviors. IGD was positively correlated to three indicators of cyberbullying in medium effect sizes (*rs* > 0, *ps* < 0.01). Moreover, the three indicators of cyberbullying were positively associated with each other in medium to large effect sizes (*rs* > 0, *ps* < 0.01). Adolescents who were involved in one online problem behavior also reported more engagement in another one. Besides, adolescents' sex and age were significantly related to the main variables as well. Therefore, they were both included as control variables in the following analyses.

**Table 1:** Descriptive statistics and correlations of main variables.

**Variable**	**Mean**	**SD**	**Skewness**	**Kurtosis**	**1**	**2**	**3**	**4**	**5**	**6**
1.Sex			0.32	−1.91						
2.Age	15.06	1.48	−0.70	−0.23	−0.07					
3.PYD	384.66	63.13	−0.23	−0.10	−0.06	0.18^**^				
4.IGD	17.92	4.64	0.55	0.34	−0.29^**^	−0.06	−0.14^**^			
5.CPV	19.17	16.33	1.52	2.14	−0.26^**^	−0.10^*^	−0.17^**^	0.32^**^		
6.CV	11.74	9.43	1.17	0.65	−0.19^**^	−0.09	−0.14^**^	0.23^**^	0.91^**^	
7.CP	7.43	8.69	1.77	2.62	−0.27^**^	−0.10^*^	−0.16^**^	0.36^**^	0.89^**^	0.62^**^

PYD, positive youth development attributes; IGD, internet gaming disorder; CPV, total cyberbullying involvement; CV, cyberbullying victimization; CP, cyberbullying perpetration.

* *p* < 0.05;

** *p* < 0.01.

### The mediating effect of PYD attributes

**Figure 1:**
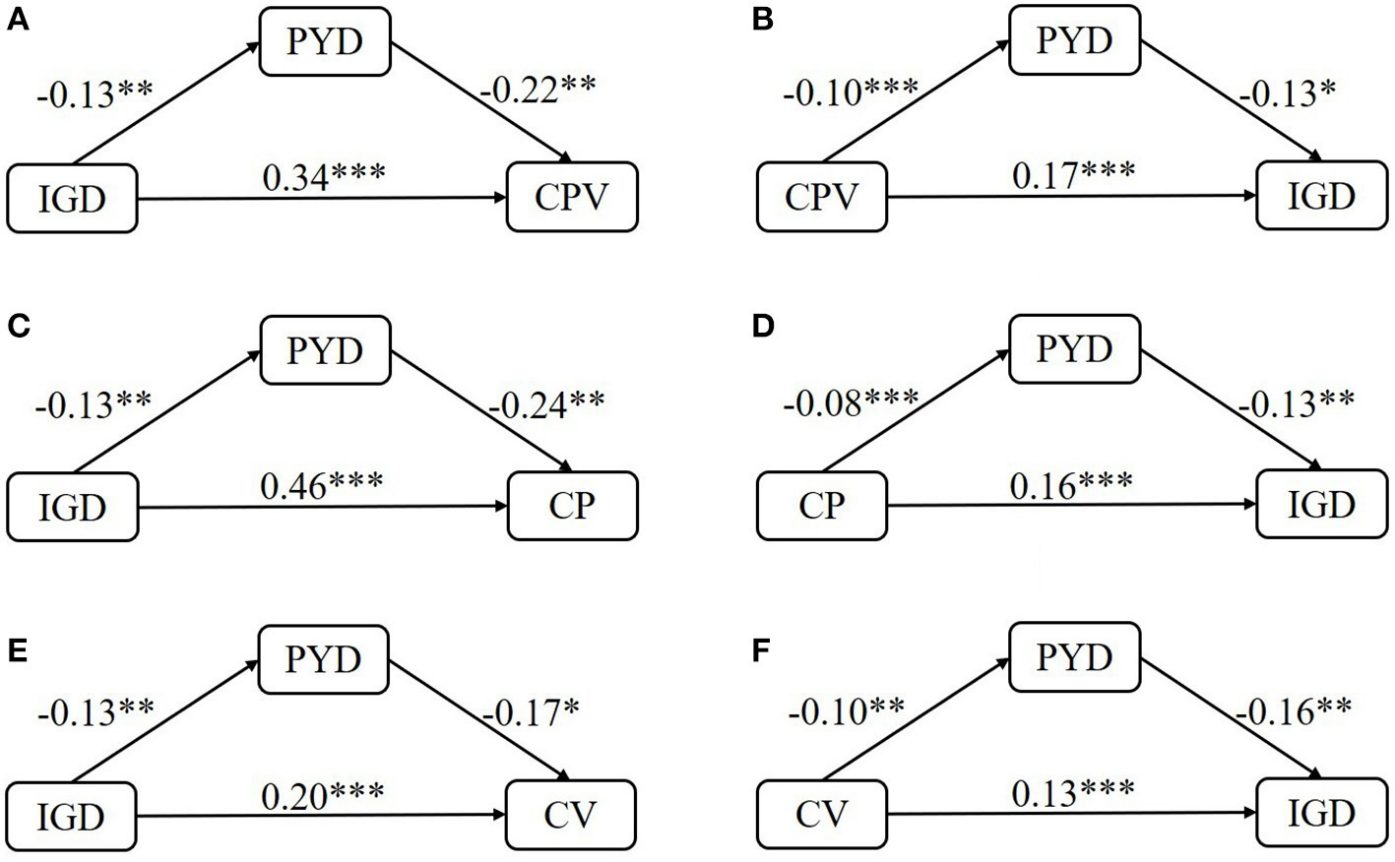
Test of the mediating effects of PYD attributes [Model **(A–F)**]. PYD, positive youth development attributes; IGD, internet gaming disorder; CPV, total cyberbullying involvement; CV, cyberbullying victimization; CP, cyberbullying perpetration. Standardized coefficients were reported. ^*^ *p* < 0.05, ^**^ *p* < 0.01, ^***^ *p* < 0.001.

To examine the possible mediating effects of PYD attributes on the association between cyberbullying/victimization and IGD, several mediating models were constructed through Model 4 in the PROCESS macro. Figure 1 displays the results of regression analyses of these models. In models A and B, the total involvement in cyberbullying positively affected IGD (β = 0.17, *p* < 0.001) and vice versa (β = 0.34, *p* < 0.001). PYD attributes negatively affected the total cyberbullying involvement (β = −0.22, *p* < 0.01) and IGD (β = −0.13, *p* < 0.05). Moreover, the total involvement in cyberbullying and IGD were significantly associated with the decrease of PYD attributes (β*s* < 0, *ps* < 0.01). In addition, the specific situations of the aforesaid relationships in various cyberbullying roles were further evaluated. In models C and D, IGD and cyberbullying perpetration could also positively affect each other in small to medium effect sizes (β*s* > 0, *ps* < 0.001). PYD attributes were significantly associated with the decreased engagement in IGD and cyberbullying perpetration, and vice versa (β*s* < 0, *ps* < 0.01). In terms of cyberbullying victimization (models E and F), it could positively affect IGD and vice versa (β*s* > 0, *ps* < 0.001). Moreover, both of them negatively affected PYD attributes with small effect sizes and vice versa (β*s* < 0, *ps* < 0.05). Overall, PYD attributes, IGD, and cyberbullying could significantly affect each other. The first, second, and third hypotheses were confirmed.

Furthermore, the 95% bootstrapped confidence intervals were estimated to evaluate the significance of the indirect effects. Table 2 shows the summary of the direct and indirect effects among the main variables. Generally, the mediating effects of PYD attributes were statistically significant in the association between IGD and total involvement in cyberbullying. Moreover, the indirect effects of PYD attributes were also significant in the relationships between IGD and specific cyberbullying behaviors (perpetration & victimization) since their 95% bootstrapped confidence intervals did not include zero. In summary, PYD attributes mediated the relationships between cyberbully and IGD, which supports the fourth hypothesis.

**Table 2:** Summary of the direct and indirect effects.

**Pathway**	**Effect**	**Boot SE**	**Boot LLCI**	**Boot ULCI**
Model A				
IGD → CPV	0.34	0.07	0.21	0.47
IGD → PYD → CPV	0.03	0.02	0.004	0.07
Model B				
CPV → IGD	0.17	0.03	0.11	0.24
CPV → PYD → IGD	0.01	0.01	0.001	0.03
Model C				
IGD → CP	0.46	0.08	0.31	0.62
IGD → PYD → CP	0.03	0.02	0.004	0.08
Model D				
CP → IGD	0.16	0.03	0.11	0.22
CP → PYD → IGD	0.01	0.01	0.001	0.03
Model E				
IGD → CV	0.20	0.06	0.09	0.32
IGD → PYD → CV	0.02	0.01	0.002	0.05
Model F				
CV → IGD	0.13	0.04	0.06	0.21
CV → PYD → IGD	0.02	0.01	0.001	0.04

PYD, positive youth development attributes; IGD, internet gaming disorder; CPV, total cyberbullying involvement; CV, cyberbullying victimization; CP, cyberbullying perpetration. Standardized regression coefficients were reported. Bootstrap sample size, 5000. CI, 95% confidence interval; LL, low limit; UL, upper limit.

## Discussion

According to the neo-ecological theory, young people's growth is influenced by both the physical and virtual environment (30). As digital natives, they enjoy the convenience and benefits of the internet but also suffer from unique developmental problems of this age, such as cyberbullying and IGD. In China, the internet and mobile devices are developing rapidly, which makes it more convenient and easier for teenagers to access the internet and play online games. To a certain degree, this also increases the risk of these developmental problems in adolescence. Fortunately, the Chinese government attaches great importance to the healthy development of children and adolescents, especially the prevention and intervention of developmental problems. In response to this policy call and considering that these online behavioral problems have high prevalence and critical adverse effects (2, 5, 7, 8), this study attempts to figure out protective factors that could significantly prevent adolescents from these problems. Theoretical and empirical evidence suggests that PYD attributes are tightly linked to cyberbullying and IGD (20, 28, 34, 35), but the underlying mechanism is still unknown. Thus, the current study investigated the interactions between cyberbullying and IGD as well as the buffering effects of PYD attributes among Chinese adolescents. Generally, the current hypotheses were confirmed in the present sample.

As expected, results supported the first hypothesis that, after controlling adolescents' sex and age, three indicators of cyberbullying could positively affect IGD and vice versa. Adolescents who experienced IGD reported more engagement in cyberbullying, and vice versa. This finding expands existing literature on the association between cyberbullying and IGD by directly revealing the interactions between them (17–19). Also, this finding enriched the literature about the relationship between aggressive behaviors and addictive behaviors (14–16). Besides, this finding provided empirical evidence for the developmental cascade theory through confirming the cascading effect between cyberbullying and IGD (13). If not intervened in time, experience with one online behavioral problem may increase the risk of engaging in another. Apart from the direct influence, involvement in one problem behavior may also increase the risks of engagement in another one indirectly. For instance, Liu et al. (16) indicated that cyberbullying victimization would lead to increased depression, which could increase the risk of internet addiction. Thus, more attention should be paid to the interactions in online problematic behaviors and investigating them from a comprehensive perspective.

Regarding the relationship between PYD attributes and online behavioral problems, the current results indicated the second hypothesis that PYD attributes had negative cumulative effects on cyberbullying and IGD. Adolescents who had more PYD attributes were less likely to suffer from cyberbullying and IGD. This observation is in line with the shared core assumption of PYD theory that the more positive attributes one has, the fewer negative outcomes he or she will develop (20). And this finding enriched the previous studies by directly confirming the cumulative effects of 15 positive attributes on cyberbullying (34, 35, 37) and empirically responded to Schiamberg et al.'s advocacy (31) to incorporate PYD theory to investigate bullying behaviors. Additionally, this finding echoes the studies of Xiang et al. (28) and Gan et al. (38) which revealed the cumulative effects of various developmental assets on reducing Chinese adolescents' IGD. And this current finding also expanded the indirect evidence on relation between several positive attributes and IGD, as well as other forms of addiction (41–44). More interestingly, the results implied that cyberbullying and IGD negatively predicted PYD attributes, which supported the third hypothesis. Adolescents who experienced online problematic behaviors reported fewer perceived positive attributes. This phenomenon may be explained by the conservation of resources theory, which claims the interactions between risks and protectors (53). Experience in cyberbullying and IGD can be considered as potential risks threatening individual healthy development, and positive attributes are protectors. So, limited PYD resources automatically offset the adverse impact of these risks to a certain degree, which leads to the resources loss.

Another important finding is that PYD attributes mediated the relationship between cyberbullying and IGD, which is in favor of the fourth hypothesis. Adolescents' PYD resources could assist in buffering the cascading effect between these two online problem behaviors. To the best of our knowledge, this is the first study proving this association between PYD attributes and developmental problems. Similar to prior literature (43, 44), this finding demonstrated the protective effect of PYD attributes in a digital context. It also expanded prior studies concerning the mediation of PYD attributes (46–48). Furthermore, the current finding is consistent with the risk-buffering model (45), which implies that PYD attributes might alleviate the risk linkage between cyberbullying and IGD. And the mediating role of PYD attributes also proved the indirect interaction between cyberbullying and IGD mentioned above. That is, adolescents who experience one behavioral problem may spend positive resources to offset the negative impact of this experience, which will lead to resource loss or the perception of fewer PYD attributes. Thus, it indirectly increases the risks of engaging in another developmental problem.

Overall, these findings have several theoretical and practical implications. Initially, cyberbullying and IGD may share a cascading effect and will result in cumulative damage. This finding reminds us of the importance of timely intervention in these behavioral problems. Parents and teachers have to monitor teenagers' use of the internet and be vigilant against online problematic behaviors. Clinical psychologists and practitioners should design more effective measures to reduce or prevent adolescents' cyberbullying and IGD. Furthermore, the predictive and bidirectional mediating effects of PYD attributes on cyberbullying and IGD remind people to notice the function of positive factors, which provides a new approach to prevent or intervene in online problem behaviors. Therefore, comprehensive measures should be taken to guide young people to develop more positive personal and social resources. Fortunately, there are mature approaches for adolescents to improve these attributes and many pioneering studies to refer to. For example, a PYD program in urban after-school settings reported that, compared to those in the control group, adolescents receiving the interventions exhibited significantly lower substance use 1 year later (54). Gavin et al.'s review (55) found that adolescents who participated in PYD programs perceived improvement in PYD attributes and developed a higher level of sexual and reproductive health. In China, a community-based PYD program was conducted on junior high school students, and the post-test results showed significant increases in 15 PYD attributes compared with baseline levels (56). Recently, a school-based PYD program suggested that experimental group students perceived greater improvement in PYD attributes and a decline in delinquency than did control group students (57). In addition to these projects, there are numerous evidence-based formats of PYD programs to adopt to assist young people to improve positive attributes, including camp, wilderness adventure, sports, art, music, and mentoring (58).

Although this study makes contributions to related literature and practice, several limitations in the current study have to be considered. First, this study only recruited a sample of Chinese adolescents, which limits the generalizability of the current findings. Future studies may consider including more samples from various cultures to explore the relation between PYD attributes and online problem behaviors. Second, the dataset is collected through self-reported questionnaires, which cannot avoid the social expectation effect. Future studies are encouraged to use teacher/parent-reported questionnaires or the peer nomination method to collect data on negative developmental outcomes. Third, this study adopts a cross-sectional design to examine the mediating effect of PYD attributes. It cannot obtain a potential causal relationship among variables. Moreover, it should be noted that the effect sizes of the mediation are small. So, scholars may employ longitudinal design to reexamine the mediation of PYD attributes between the two online problem behaviors. Fourth, the present study only focuses on the whole situation of IGD and its association with cyberbullying (perpetration & victimization). However, research has demonstrated that playing online games, especially violent games, might increase the risk of cyberbullying among adolescents (59, 60). Moreover, cyberbullying includes different roles, such as cyberbully, cyber-victim, cyberbully-victim, and bystander (61, 62). Therefore, future research may investigate the relationships between various types of IGD and different roles of cyberbullying to deepen the understanding of the aforesaid association *via* a more microscopic view. Fifth, the current study adopts the 15 PYD attributes model to explore the protective role in online problem behaviors, which concentrates more on personal resources. As reviewed, there are other positive factors from the environmental system, such as the external resources in developmental assets (21). Hence, future research is encouraged to evaluate the protection of both internal and external resources in adolescents' online problematic behaviors.

In conclusion, the present study attempts to prevent digital natives from unique online behavioral problems (cyberbullying and IGD) from the PYD perspective. The current findings reveal the relationship between cyberbullying and IGD, as well as the mediating role of PYD attributes in Chinese youth. Positive personal resources function effectively in adolescents' online behavioral problems, which contributes to the related prevention and intervention in cyberbullying and IGD in a sense.

## Data availability statement

The raw data supporting the conclusions of this article will be made available by the authors, without undue reservation.

## Ethics statement

The studies involving human participants were reviewed and approved by the Research Ethics Committee of the College of Education and Sports Sciences at Yangtze University. Written informed consent to participate in this study was provided by the participants' legal guardian/next of kin.

## Author contributions

Y-HZ and XG contributed to conception and design of the study. Y-NZ, ML, and XJ collected the dataset. G-XX and K-NQ performed the statistical analysis. G-XX drafted and revised the manuscript. All authors contributed to study and approved the submitted version.

## Funding

This research was supported by Youth project of the Ministry of Education in 2020 in the 13th Five-Year Plan of National Education Science: The heterogeneous developmental trajectory of internet gaming disorder in adolescents: The combined action of parenting environment and genetic polymorphism (grant number: EBA200391).

## Conflict of interest

The authors declare that the research was conducted in the absence of any commercial or financial relationships that could be construed as a potential conflict of interest.

## Publisher's note

All claims expressed in this article are solely those of the authors and do not necessarily represent those of their affiliated organizations, or those of the publisher, the editors and the reviewers. Any product that may be evaluated in this article, or claim that may be made by its manufacturer, is not guaranteed or endorsed by the publisher.
